# Monomania—Phrenology

**Published:** 1831

**Authors:** 


### Monomania—Phrenology.

In a late clinical lecture at St. Thomas's Hospital, Dr. Elliotson alluded to a curious case of monomania, which we shall here notice.

The patient was a female, 31 years of age, who had been admitted for a nervous affection, but who was soon found to be monomaniacal. The propensity was to injure some part or other of her body, by internal muscular efforts, and not by attempts at cutting or maiming. The part of the body that fell under the monomaniacal displeasure was never long the same, but varied from hour to hour—commencing in one part whenever it had ceased in another, so that she had no respite from this harassing propensity. Her judgment on all points was good—she had no hallucination—and was conscious of the unnatural impulse which perpetually led to injure her own body. There was no reason to suspect a feigned disease. She had head-ache, drowsiness, sense of pressure on the head, as well as that of "opening and shutting," to use her own expression, behind the ears and round the back part of the head. Her breath was so offensive that it was impossible to inhale it without sickness. Her tongue was very much furred. She was sleepless. Her occupation had been that of teaching children, and working at her needle. Dr. Elliotson considered this case as one illustrating the doctrines of phrenology—namely, that it was a case where the organ of destructiveness was in an excited state, and consequently where the "instinct carnassier," as Spurzheim would term it, was strongly called forth. Here, indeed, it was not a homicidal but a suicidal propensity that existed; yet the nature of both is the same. The peculiarity in the present case, which attracted attention, was the pain and throbbing just over each ear, and extending posteriorly round the head. Every gentleman who attended to the case, was witness of this phenomenon. "This (says Dr. E.) was a very striking phrenological fact," and he goes on to state his belief in the truth of the general doctrines of phrenology. "I have examined (says he) the subject of phrenology most carefully and unremittingly, and have seldom allowed a day to pass without making some observations upon it; and, after thus examining it for ten or twelve years, I am more and more satisfied of the general truth of what Dr. Gall has announced."

"With respect, however, to the *treatment* of this woman, our patient, I considered that an inflammatory affection of that part of the head, was the cause of the pain and morbid desire which she experienced. I cupped her behind the ears to twelve ounces, and gave her calomel five grains, twice a day, and put her on low diet. Leeches were again and again applied to that part. She was admitted on the 18th of January, and 20 leeches were applied to the seat of the pain every day until the 8th of February. From that time they were applied every other day for a fortnight. Her mouth soon became tender, and as that took place, her tongue became clean, and her breath ceased to stink. At last it began to smell of mercury, but the odour was quite changed in its character, and was supportable. She twisted her head less and less, and she slept more. She was observed to sleep several hours in the night. The pain in the head left her, and she now felt relieved from the disposition to strain and injure herself. She was a deformed woman, and the lungs and heart had hardly any room to play. She was subject to more or less bronchitis, and was seized with an acute attack from being placed near a window, and it was necessary, on account of this affection of the chest, to bleed her. She was bled to six ounces, recovered from the bronchitis, was now really well, and was to be presented on the following Thursday. On the Saturday, however, being in the ward, just as usual, she was seized, I understand, (for I had left the hospital,) with pain in the abdomen, and in a few minutes died. The friends came for the body before the inspection could be completed. The head was examined, and also the chest, but nothing morbid was found in either, except the adhesion of the dura mater to the brain above the meatus auditorius externus. On account of the friends waiting for the body, the examination of the abdomen was not proceeded with, and therefore the cause of death is shut up in mystery. Whether she died from a rupture of any-thing in the abdomen, I do not know, but she was seized with pain there, and in two minutes was dead. I have no idea at all of the nature of the cause of her death, although I am quite satisfied that it was in the abdomen."

Dr. Elliotson mentions some curious cases of monomania, and among others that of a lady who was constantly harassed with the dread of pollution from filth in the streets or houses. In another individual, the dread of typhous contagion was predominant, so that she would not allow her medical attendant to come in contact with her. We could add half a hundred instances of similar kinds from our own experience; but the narrative would be tiresome and useless. One of these is a lady who dreads the contagion of hydrophobia, and dare not walk the streets lest she should encounter a mad animal, or the saliva of one, which to her is as dreadful as the bite.

